# Development of Forsythia Essential Oil Microemulsions: Effects of Surfactants on Stability and Antibacterial Activity

**DOI:** 10.1021/acsomega.5c00059

**Published:** 2025-08-08

**Authors:** Hanhang Yang, Ruowen Li, Hao Ma, Lingtao Tian, Jingchen Yan, Gang Chen, Zhifeng Zhang

**Affiliations:** † Faculty of Chinses Medicine, State Key Laboratories for Quality Research in Chinese Medicines, 58816 Macau University of Science and Technology, Avenida Padre Tomas Pereira, Taipa, Macau 999078, China; ‡ Center for Drug Research and Development, 71237 Guangdong Pharmaceutical University, Guangdong 510006, China

## Abstract

This study developed a stable Forsythia essential oil (FEO) microemulsion (FEO-ME) using a self-microemulsifying drug delivery system (SMEDDS). The FEO-ME effectively reduced the volatility of essential oil and inhibited bioactive component degradation. By constructing pseudoternary phase diagrams, the optimal formulation was identified, utilizing the surfactant EL-40 and cosurfactant ethanol. The resulting microemulsion exhibited an average droplet size of 21.58 nm and an encapsulation efficiency of 85.6%. FEO-ME demonstrated excellent storage stability over 15 days, with minimal changes in droplet size and polydispersity index (PDI), ensuring long-term reliability. Additionally, antibacterial tests revealed enhanced efficacy against *Escherichia coli* compared to raw FEO. These findings highlight the potential of FEO-ME to enhance the bioactivity, stability, and applicability of essential oils with promising applications in food preservation, pharmaceuticals, and other industries.

## Graphical abstract

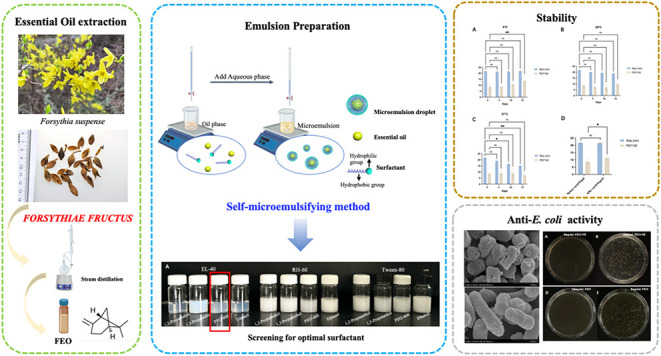

## 1 Introduction

*Forsythia suspensa* is a valuable plant widely distributed across China, Korea, Japan, and parts of Europe, known for both edible and medicinal properties.^1^ Forsythia essential oil (FEO) is a distillate derived from the fruit of Forsythia, primarily composed of α-pinene, β-pinene, and limonene.^2^ These components confer FEO with potent antimicrobial, anti-inflammatory, antioxidant, and neuroprotective activities.^3–5^ Previous studies have demonstrated that FEO exhibits significant antibacterial effects against foodborne pathogens, including *Escherichia coli* (*E. coli*) and *Staphylococcus aureus* (*S. aureus*).^1,6^ This makes FEO a promising natural food preservative capable of replacing chemical preservatives that pose health risks. Cheng et al.^7^ utilized FEO in antimicrobial films, which demonstrated enhanced antioxidant and antibacterial properties, thus extending the shelf life of meat products. In the pharmaceutical field, the overuse of antibiotics has led to bacterial resistance.^8^ Essential oil can serve as synergistic adjuvants when combined with antibiotics, lowering the minimum inhibitory concentration (MIC) of antibiotics through mechanisms such as membrane disruption.^9–12^ This synergy provides a viable strategy to curb antibiotic overuse.

However, the application of essential oils is limited by their high volatility, low water solubility, and degradation into toxic products under environmental stress, such as heat, light, and oxygen.^13–15^ For example, D-limonene, a major component of FEO, becomes a sensitizer upon oxidation.^16,17^ These factors make the manipulation and handling of these compounds difficult and are significant drawbacks to the commercialization of formulations containing such molecules.

To address these challenges, a promising approach involves emulsifying essential oil to form microemulsions,^18,19^ nanoemulsions,^20–22^ and pickering emulsions.^23,24^ Microemulsions, defined by droplet sizes between 10 and 100 nm,^25^ are characterized by their thermodynamic stability, isotropic, and transparency,^26,27^ offering several advantages over other emulsions. These advantages include enhancing water solubility and thermodynamic stability of essential oil, increasing their antimicrobial activity, reducing the volatility of active ingredients, preventing oxidation, and minimizing skin irritation and toxicity.^28–32^ Additionally, microemulsions have a smaller droplet size and longer shelf life compared to other emulsions.^33,34^

While conventional essential oil encapsulation predominantly relies on high-energy methods (*e.g*., ultrasound, high-pressure homogenization),^35–37^ which can lead to the volatilization of essential oil and degradation of active ingredients due to localized high temperatures. However, these methods are energy-intensive and may compromise the stability of the active compounds. In contrast, self-microemulsifying systems form microemulsions through the spontaneous assembly of surfactants under mild stirring, without the need for high-energy inputs.^38,39^ This approach avoids the risks associated with high-energy methods, helping preserve the stability of essential oil and enabling long-term storage.

While the use of microemulsions in essential oil encapsulation has been explored, the application of Forsythia essential oil (FEO) and its microemulsions (FEO-ME) have received limited attention. Additionally, most studies focus on high-energy techniques,^7,40^ and the use of self-microemulsifying systems for FEO encapsulation has not been extensively studied. This study addresses this gap by preparing Forsythia essential oil microemulsions (FEO-ME) using self-microemulsifying drug delivery systems and evaluating their stability and particle size distribution through pseudoternary phase diagrams to optimize the formulation.^38^ Among 12 different surfactant combinations, a highly stable microemulsion with a high encapsulation efficiency for FEO (FEO-ME) was identified. The results show FEO microemulsion significantly enhanced the antibacterial activity of FEO against *E. coli*. This research extends the potential applications of Forsythia essential oil in food preservation and pharmaceutical formulations, providing a novel approach to using this bioactive compound.

## 2 Materials and Methods

### 2.1 Materials

*F. suspensa* [(Thunb.) Vahl (Oleaceae)] fruits were collected from Xi’an, Shanxi Province, China, and identified by Professor Zhang Zhifeng from the Faculty of Chinese Medicine at Macau University of Science and Technology. Forsythia essential oil (FEO) was extracted by steam distillation and stored at 4 °C for later experiments.

The surfactants EL-40 (Shanghai Maclin Biochemical Technology Co., Ltd., China), RH-60 (Qingdao Ke Hao Biotechnology Co., Ltd., China), and Tween-80, along with cosurfactants 1,2-propanediol, glycerol, PEG-400, and anhydrous ethanol (Shanghai Maclin Biochemical Technology Co., Ltd., China), were utilized for microemulsion preparation. Milli-Q water was used as the aqueous phase in all formulations.

### 2.2 Analysis of FEO Using Gas Chromatography–Mass Spectrometry (GC-MS)

Chromatographic separations were carried out using a 30 m × 0.25 mm × 0.25 μm fused silica capillary column (DB-5MS; J&W Scientific, Folsom, CA) on an Agilent 7890B gas chromatograph coupled with an Agilent 5977B mass spectrometer (Agilent Technologies, Santa Clara, CA). In splitless injection mode, helium was used as the carrier gas. The temperature of the column was set at 40 °C initially, ramped up to 140 °C at a rate of 5 °C/min, and then held for 5 min. Subsequently, the ambient temperature was increased to 280 °C at 10 °C/min and maintained for 1 min.

The electron impact (EI) ionization method was used for mass spectrometry, with an electron energy of 70 eV and an ion source temperature of 250 °C. Mass spectra were recorded across a scan range of 20–500 *m*/*z*. A solvent delay of 3 min was applied before the analysis. The chemical constituents of FEO were identified by comparing their mass spectra to those in the NIST 11 mass spectrometry library. The relative percentages of individual components were calculated by normalizing the peak areas.

### 2.3 Selection of Surfactants

EL-40, RH-60, and Tween 80 were selected as surfactants. 1,2-propanediol, 1,3-propanediol, PEG-400, and ethanol were used as cosurfactants. Twelve kinds of different surfactant mixtures (S_mix_) were prepared by combining the surfactants and cosurfactants in a 4:1 weight ratio. Each formulation contained 0.8 g of Forsythia essential oil (FEO) as the oil phase, 2.5 g of the surfactant mixture was used, and 6.7 g of Milli-Q water was added to each formulation. The appearance and droplet size of the emulsions were used to screen for the optimal surfactant.

### 2.4 Construction of Pseudoternary Diagrams (PTDs)

Pseudoternary diagrams were prepared according to the method described by previous studies.^41,42^ The PTD was constructed using the oil phase, aqueous phase, and surfactant mixture. After setting the surfactant to cosurfactant ratio, the mixtures were combined with different amounts of oil phase, and titrated with water at room temperature. During titration, the appearance of the emulsion was visually monitored, and the amount of water added when the emulsion became transparent was recorded. The phase diagram was constructed based on the weight ratios of the three components. The size of the microemulsion region was compared by direct observation.

Using FEO as the oil phase and EL-40 as the surfactant, three different cosurfactants (1,2-propanediol, PEG-400, and ethanol) were tested for their ability to form microemulsions. The ratio of the surfactant to the cosurfactant was 4:1. FEO was mixed with the surfactant blend in oil-to-surfactant weight ratios of 9:1, 8:2, 7:3, 6:4, 5:5, 4:6, 3:7, 2:8, and 1:9. The mixture was stirred continuously at 500 rpm, and Milli-Q water was added at a rate of 1 mL/min. The amount of water required to reach transparency was recorded. Based on the obtained data, a pseudoternary phase diagram was constructed, and the cosurfactant that resulted in the largest microemulsion region was identified. Subsequently, microemulsion areas for different mass ratios of surfactant to cosurfactant were mapped to determine the optimal surfactant ratio.

### 2.5 Microemulsions Characterization

#### 2.5.1 Identification of the Type of FEO-ME

The type of FEO-ME was determined by using a staining method. At room temperature (25 °C), a water-soluble methylene blue solution (5 mg/mL) and oil-soluble Sudan III solution (prepared by dissolving 0.1 g in 10 mL of ethanol and mixing with 10 mL of glycerin) were added dropwise to the microemulsion. The diffusion rates of the dyes were observed to determine whether the microemulsion was an O/W or W/O.

#### 2.5.2 Droplet size, PDI, ζ-Potential Measurements, and PH

With a Zetasizer Nano-ZS90 analyzer (Malvern Instruments Co., Ltd., Worcestershire, U.K.), the average droplet size, PDI, and ζ-Potential of FEO-ME were ascertained. Before measurement, all of the samples were diluted 50-fold with Milli-Q water. The equilibrium time was set at 120 s, and the temperature was 25 °C. Three readings per sample were averaged and reported. The pH value of the microemulsion was measured at room temperature using a pH meter (ST20, OHAUS Corporation).

#### 2.5.3 Morphology of FEO-ME

The morphology of FEO-ME was observed by a transmission electron microscope (FEI TECNAI G2 12, Thermo Fisher Scientific) operating at an accelerating voltage of 100 kV. To observe the size and shape of the nanodroplets, FEO-ME was diluted 100 times with Milli-Q water. A 10 μL aliquot of the diluted sample was placed on the copper grid, which was then left for 10 min. Afterward, excess liquid was absorbed by using filter paper. The grids were stained with 10 μL of 3% (w/v) uranyl acetate for 1–3 min, and excess staining liquid was absorbed with filter paper for negative staining. The samples were randomly imaged under transmission electron microscopy (TEM) at an accelerating voltage of 100 kV.

#### 2.5.4 Storage Stability

FEO-ME were placed in covered centrifuge tubes and stored at 4, 25, and 37 °C for 30 days. Droplet size and PDI were measured every 5 days to monitor changes over time.

#### 2.5.5 Centrifugal Stability

The centrifugal stability of FEO-ME was assessed by diluting the microemulsions 2-fold with distilled water and centrifuging at 3803×*g* for 15 min. Samples were collected from the bottom layer, and the absorbance at 280 nm was measured using a spectrophotometer. The centrifugation stability constant (*K*_e_) was calculated to quantify the stability.

$$K_{e}={\frac{\left(A_{0}-A\right)}{A_{0}}}^{*}\times 100\%$$

#### 2.5.6 Determination of Encapsulation Efficiency

Add 2 mL of microemulsion to 5 mL of *n*-hexane, shake for 1 min, allow phase separation, and take the upper organic phase for measurement of its absorbance value to calculate the unloaded oil content. Take 4 mL of microemulsion and sonicate at 400W for 20 min to break the emulsion, add 10 mL of *n*-hexane, shake for 1 min, allow phase separation, and take the upper organic phase for absorbance measurement to calculate the loaded content. The encapsulation efficiency was calculated according to the formula

$$e\mathrm{ncapsulation}\;e\mathrm{fficiency}\left(\%\right)=\frac{t\mathrm{otal}\;o\mathrm{il}-u\mathrm{npackaged oil}}{t\mathrm{otal Oil}}\times 100\%$$

### 2.6 *In Vitro* Investigation on *Escherichia coli* (*E. coli*)

#### 2.6.1 Agar Diffusion Method

The antibacterial activity of Forsythia essential oil microemulsions (FEO-ME) against *E. coli* (ATCC 25922) was evaluated using the Oxford cup method, according to the Clinical Laboratory Standards Institute (CLSI) recommendations^43^ with some modifications. *E. coli* suspension (1 × 10^8^ CFU/mL) was spread on NA agar plates using a cotton swab, and FEO-ME was added to sterilized cups placed on the agar surface. The inhibition zone diameter was measured after 24 h of incubation at 37 °C. Four groups were set up for the experiment: 1. FEO-ME group, 2. FEO group, 3. Blank control (Milli-Q water) 4. Blank microemulsion group: prepared identically to FEO-ME but replacing FEO with Soybean oil (nonantimicrobial carrier), maintaining surfactant/cosurfactant ratio (4:1).

#### 2.6.2 Minimum Inhibit Concentration (MIC) and Minimum Bactericidal Concentration (MBC)

MIC and MBC values were determined using 2-fold dilutions, as described previously^43^ with some modifications. Each well was inoculated with *E. coli* suspension (50 μL, 1 × 10^6^ CFU/mL), and incubation was performed at 37 °C for 16–18 h. MIC was the lowest concentration without visible growth. MBC was determined by plating samples from nongrowth wells onto NA plates and incubating for 16–18 h. The concentration of FEO-ME in the culture without microorganism growth was confirmed as MBC.

#### 2.6.3 Scanning Electron Microscopy (SEM) Test

Bacterial morphology was examined using SEM, as described previously^44^ with some modifications.10 mL of *E. coli* suspensions with 1 × 10^6^ CFU/mL were incubated for 24 h at 37 °C. After centrifuging at 4500 rpm for 7 min, equal parts of FEO and FEO-ME were extracted from each sample. After incubation for 24 h at 37 °C, the samples were centrifuged. Bacteria were fixed at 4 °C overnight using glutaraldehyde (2.5/100 mL) after two washes with phosphate-buffered saline (0.1 mol/L). Next, increasing ethanol concentrations (50, 70, 100, and 100%) were used to dry the bacteria. After dehydration, the samples were removed from 100% ethanol and subjected to critical point drying for 1 h. The samples were then fixed on the sample stage with conductive adhesive tape for gold sputtering. The bacterial morphology was examined by using SEM at a voltage of 5.0 kV. As a blank control, LB-treated bacteria were employed.

#### 2.6.4 Statistical Analysis

Data are expressed as means ± the standard deviation (SD) from three independent replicates. For comparisons of mean particle size and PDI values, one-way analysis of variance (ANOVA) was performed to determine whether there were significant differences between groups. When ANOVA showed significant results, Tukey’s post hoc test was applied to compare individual groups. A *p*-value of less than 0.05 was considered statistically significant. All statistical analyses were conducted using GraphPad Prism version 9.0 (GraphPad Software Inc., San Diego, CA). For plotting pseudoternary phase diagrams, OriginPro version 10.0 (OriginLab) was used.

## 3 Result and Discussion

### 3.1 GC-MS Detection Results

The typical total ion chromatogram produced under the applied extraction conditions is shown in Figure 1. A total of 29 compounds were identified based on their mass spectra, which were compared with the standard values described in the literature and the mass spectrometry reference library. The relative concentrations of each peak, are summarized in Table 1. The results reveal the following order of the main compounds by concentration: Bicyclo[3.1.1]­heptane, 6,6-dimethyl-2-methylene-, (1S) (45.7%), α-Pinene (17.7%), 3-Cyclohexen-1-ol, 4-methyl-1-(1-methylethyl)- (7.8%), Bicyclo[3.1.0]­hex-2-ene, 2-methyl-5-(1-methylethyl)- (5.2%), D-Limonene (4.9%), γ-Terpinene (3.4%), β-Myrcene (2.5%), Cyclohexene, 1-methyl-4-(1-methylethylidene)- (2.2%), Camphene (1.6%), Benzene, 1-methyl-4-(1-methylethyl)- (1.5%), 3-Cyclohexene-1-methanol, α,α-4-trimethyl-1- (1.4%), Cyclohexene, 3-methyl-6-(1-methylethylidene)- (1.0%), (1R)-(−)-Myrtenal (1.0%), trans-Pinocarveol (0.9%), Pinocarvone (0.7%), 2-Cyclohexen-1-ol, 1-methyl-4-(1-methylethyl)-, cis- (0.3%).

**Figure 1:**
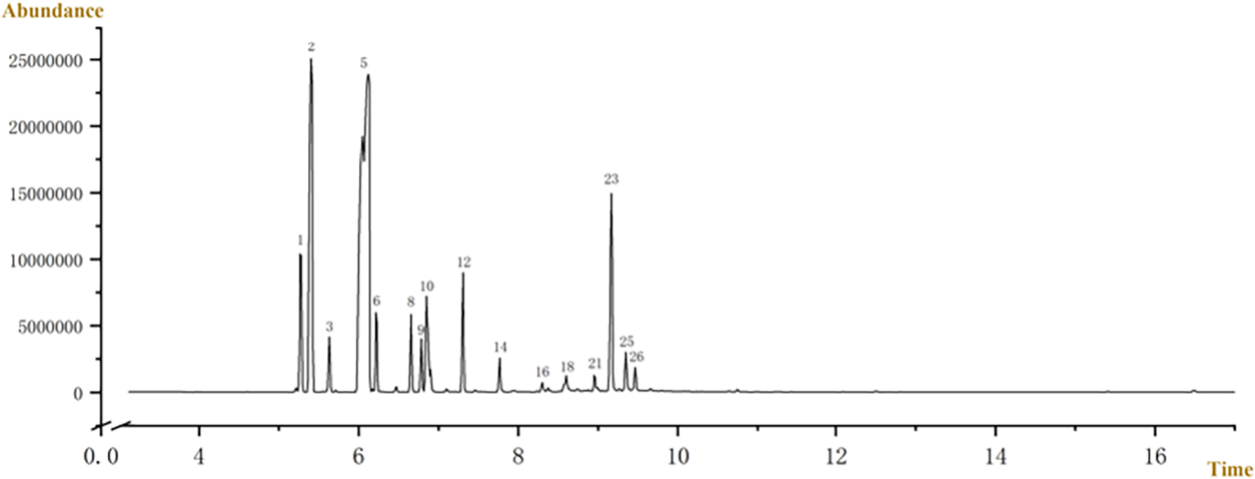
Total ion current chromatogram of the FEO.

**Table 1:** Main Volatile Components in FEO

no.	retention time(min)	semiquantitative result	compounds
**5**	6.121	45.7%	bicyclo[3.1.1]heptane, 6,6-dimethyl-2-methylene-, (1S)-
**2**	5.405	17.7%	α-pinene
**23**	9.166	7.8%	3-cyclohexen-1-ol, 4-methyl-1-(1- methylethyl)-
**1**	5.267	5.2%	bicyclo[3.1.0]hex-2-ene, 2-methyl-5-(1-methylethyl)-
**10**	6.848	4.9%	D-Limonene
**12**	7.307	3.4%	γ-Terpinene
**6**	6.217	2.5%	β-Myrcene
**8**	6.655	2.2%	cyclohexene, 1-methyl-4-(1-methylethylidene)-
**3**	5.630	1.6%	camphene
**9**	6.784	1.5%	benzene,1-methyl-4-(1-methylethyl)-
**25**	9.348	1.4%	3-Cyclohexene-1-methanol, α,α-4-trimethyl-1-
**14**	7.766	1.0%	cyclohexene, 3-methyl-6-(1-methylethylidene)-
**26**	9.465	1.0%	(1R)-(−)-Myrtenal (1.0%)
**18**	8.600	0.9%	trans-Pinocarveol
**21**	8.952	0.7%	pinocarvone
**16**	8.301	0.3%	2-cyclohexen-1-ol, 1-methyl-4-(1-methylethyl)-, cis-

### 3.2 Formulation Development

#### 3.2.1 Selection of Surfactants

By comparing the combinations of 12 surfactants and cosurfactants and cosurfactants (Figure 2A), it was observed that EL-40, when used as a surfactant, produced a more transparent microemulsion compared to RH-60 and Tween-80, with particle sizes below 100 nm (Figure 2B). When used as cosurfactants, 1,2-propanediol, PEG-400, and ethanol resulted in smaller particle sizes compared to glycerol. Since microemulsions with smaller particle sizes tend to exhibit better stability, EL-40 was selected as the surfactant for FEO, while further screening of cosurfactant will be focused on 1,2-propanediol, PEG-400, and ethanol.

**Figure 2:**
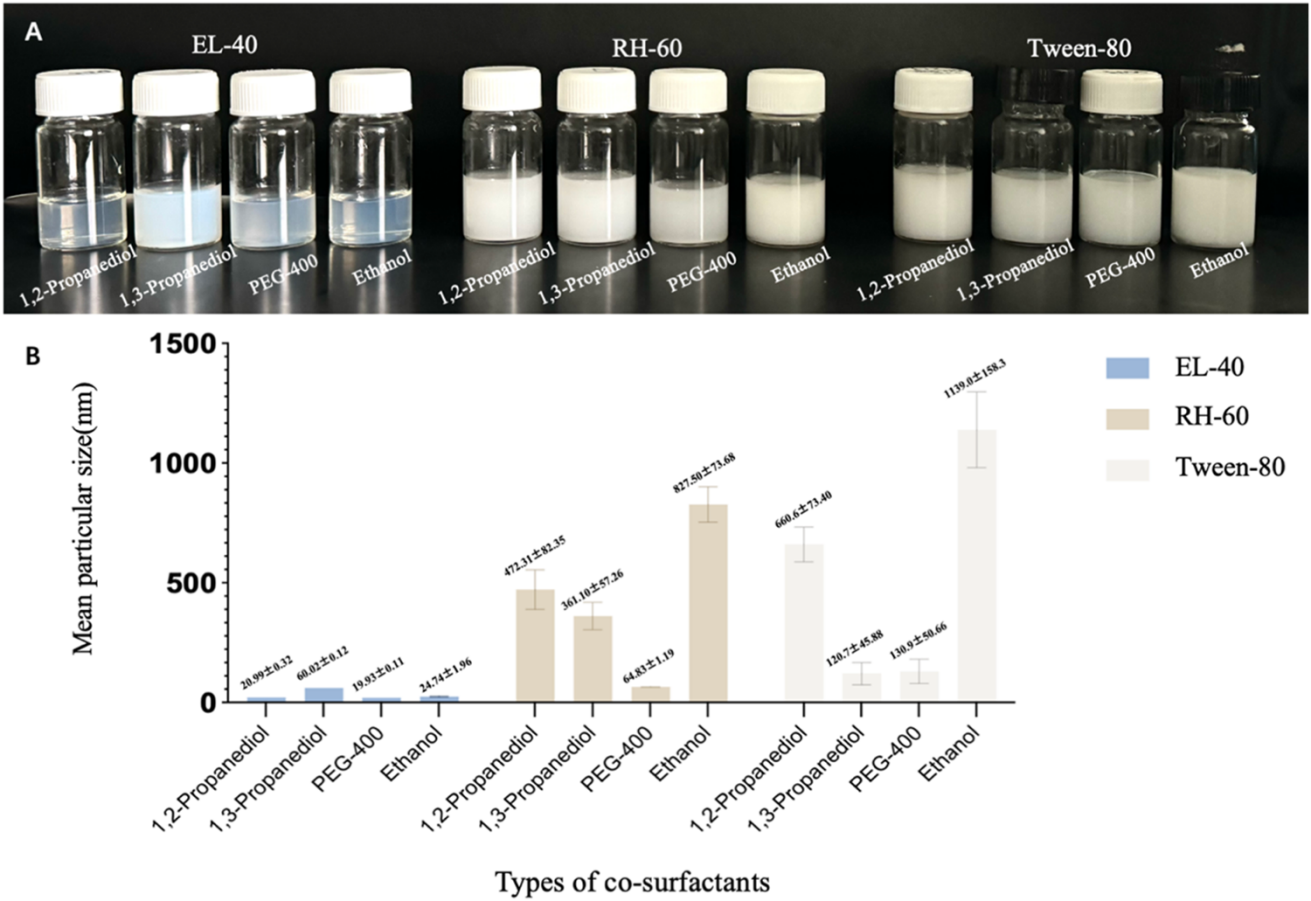
Optimal surfactant was selected from 12 different surfactant-co-surfactant combinations. (A) Appearance of 12 different FEO-MEs formulated with 3 surfactants (EL-40, RH-60, Tween-80) and 4 cosurfactants (1,2-propanediol, PEG-400, and ethanol). (B) Mean particle size of 12 different FEO-MEs formulated with 3 surfactants and 4 cosurfactants.

#### 3.2.2 Selection of Cosurfactants

1,2-propanediol, PEG-400, and ethanol were selected as cosurfactants for the experiment, and a PTD was constructed. The results shown in Figure 3A, B, and 3C demonstrate that the PTD area is larger when ethanol is used as the cosurfactant, indicating its suitability for forming a larger microemulsion region.

**Figure 3:**
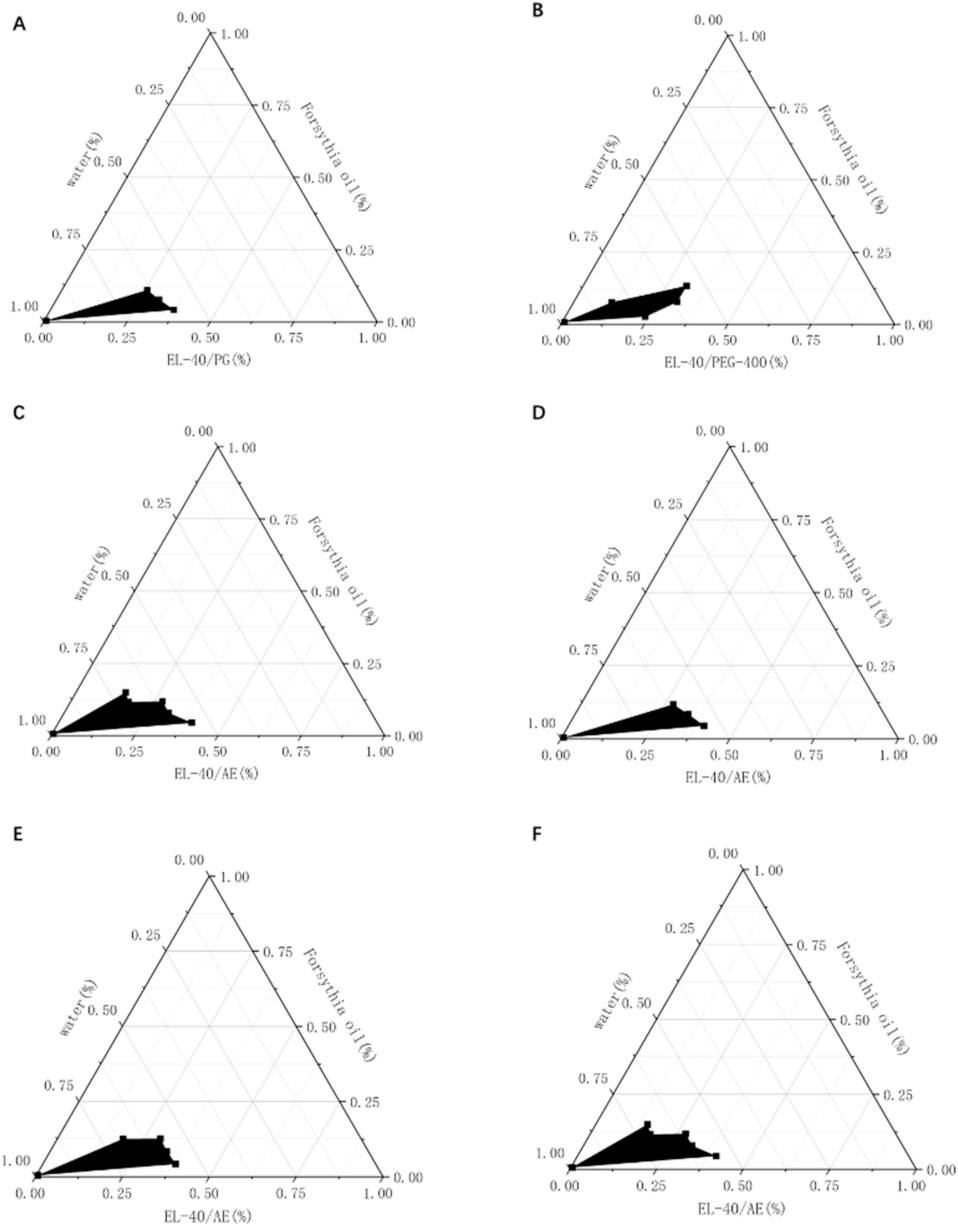
PTDs were constructed using EL-40 as the surfactant, in a 4:1 ratio with three different cosurfactants and Forsythia essential oil as the oil phase (A, B, C). (A) PTDs using 1,2-propanediol as the cosurfactant. (B) PTDs using PEG-400 as the cosurfactant. 3: PTDs using ethanol as the cosurfactant. The optimal weight ratio of surfactant and cosurfactant was determined using the PTDs of EL-40 as a surfactant, ethanol as a cosurfactant, and Forsythia essential oil as the oil phase (D, E, F). (D) Surfactant to cosurfactant ratio of 2:1. (E) Surfactant to cosurfactant ratio of 3:1. (F) Surfactant to cosurfactant ratio of 4:1. The dark region indicates the area where microemulsion forms.

#### 3.2.3 Selection of Surfactants/Cosurfactants Ratios

The PTD of the investigated quaternary system (FEO/water/EL-40/ethanol) is presented in Figure 3D,3E, and 3F. Microemulsions were formed at ambient temperature. It was evident from the phase diagrams that the region of microemulsions expanded as the weight ratio of surfactants to cosurfactants increased.

#### 3.2.4 Selection of Optimal Prescriptions

Based on the PTD with a surfactant-to-cosurfactant weight ratio of 4:1, five prescriptions in the microemulsion region were selected for microemulsion preparation. Prescription 3 exhibited an average particle size of 21.58 ± 0.150 nm for FEO-ME, with a PDI of 0.087 ± 0.014 (Table 2). After 15 days of storage, the average particle size remained essentially unchanged at 18.76 ± 0.380 nm, and the PDI remained below 0.3 (0.100 ± 0.024), demonstrating high stability of the FEO-ME with a high drug-loading capacity for FEO-ME (Table 3).

**Table 2:** Particle Size, PDI, and Appearance Characteristics of Five Selected FEO-ME Prescriptions

prescription (FEO-S_mix_-water)	particle size (nm)	PDI	appearance characteristics
1(11.76:17.64:70.6)	49.76 ± 0.110	0.250 ± 0.111	clarification and transparency
2(11:27.4:61.6)	52.44 ± 0.700	0.204 ± 0.105	clarification and transparency
3(10:30:60)	21.58 ± 0.150	0.087 ± 0.014	clarification and transparency
4(8.36:31.34:60.3)	19.61 ± 0.122	0.112 ± 0.013	clarification and transparency
5(8:32:60)	18.79 ± 0.153	0.098 ± 0.015	clarification and transparency

**Table 3:** Particle Size, PDI, and Appearance Characteristics of Five Selected FEO-ME Prescriptions after 15 Days of Storage

prescription (FEO-S_mix_-water)	particle size (nm)	PDI	appearance characteristics
1(11.76:17.64:70.6)	88.50 ± 1.457	0.393 ± 0.06	emulsion layering
2(11:27.4:61.6)	34.31 ± 0.778	0.501 ± 0.021	emulsion layering
3(10:30:60)	18.76 ± 0.380	0.100 ± 0.024	clarification and transparency
4(8.36:31.34:60.3)	21.86 ± 1.136	0.231 ± 0.071	clarification and transparency.
5(8:32:60)	19.65 ± 0.095	0.243 ± 0.013	clarification and transparency

### 3.3 Characterization of the FEO-ME

#### 3.3.1 Determination of the Basic Properties of FEO-ME

The prepared FEO-ME is clear and transparent with a slight blue tint. When illuminated by a laser pen, the emulsion exhibits a completely bright pathway, as shown in Figure 4A. Upon the addition of methylene blue dye and Sudan IV dye dropwise to the emulsion, the diffusion rate of methylene blue was significantly faster than that of Sudan IV dye (Figure 4B). This observation indicates that the emulsion prepared in this study is an O/W (oil-in-water) type microemulsion.

**Figure 4:**
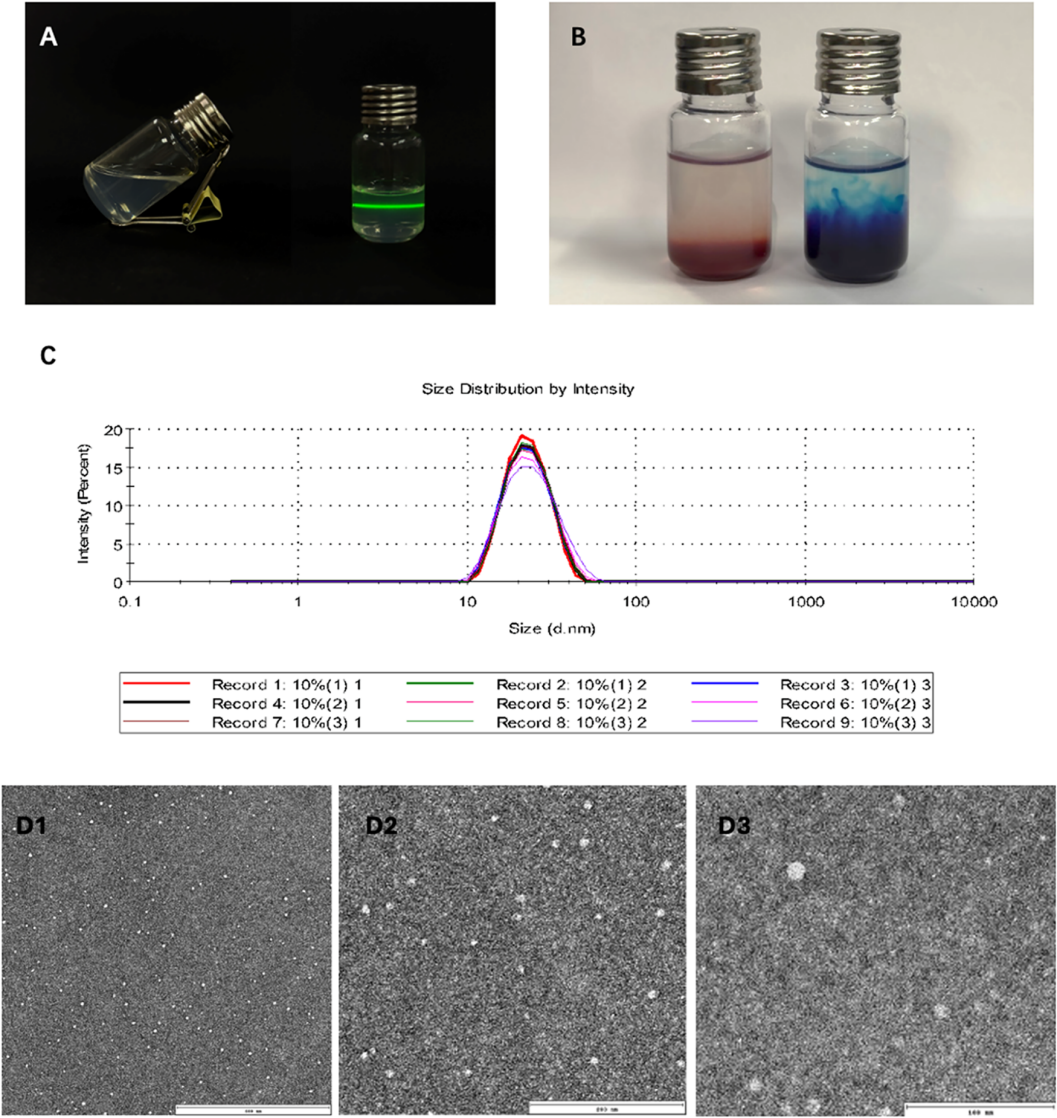
Appearance characteristics of FEO-ME, dye diffusion experiments, and basic properties. (A) Visual characteristics of FEO-ME (left: The flowability of the microemulsion; right: Tyndall effect observed during laser beam transmission through the microemulsion, confirming the presence of nanoscaled droplets). (B) Sudan III dye diffusion confirming water-in-oil (W/O) microstructure. (C) Particle size distribution of FEO-ME. (D) TEM images of FEO-ME at different scales: 500 nm­(D1), 200 nm­(D2), and 100 nm­(D3).

#### 3.3.2 Basic Characteristics of FEO-ME

Particle size distribution, ζ-potential, mean particle size, and PDI are the key characteristics of microemulsions. The average pH of the FEO-ME is 5.89, the mean particle size of FEO-ME is 21.58 ± 0.15 nm, and the PDI is 0.087 ± 0.014. The particle size distribution (Figure 4C) shows an extremely narrow peak, suggesting that the droplet size is highly uniform. TEM images (Figure 4D) revealed that FEO-ME has uniform droplet sizes, and droplets appear spherical. The average ζ-potential of FEO-ME was −9.86 ± 0.522 mV. These results provide a solid basis for further evaluation of FEO-ME stability after storage.

#### 3.3.3 Determination of Encapsulation Efficiency

The encapsulation efficiency of FEO-ME was found to be 85.6 ± 0.98%, indicating that the encapsulation method is effective and reliable.

### 3.4 Stability of FEO-ME

#### 3.4.1 Storage Stability

Droplet size and PDI of the different microemulsions were measured after 15 days of storage. The results are shown in Figure 5(A–C). After 15 days, no significant changes were observed in these parameters. The PDI of FEO-ME, prepared by our optimized method, remained below 0.1, which minimized the likelihood of Ostwald ripening. Therefore, FEO-ME demonstrated stability over long-term storage at various temperatures.

**Figure 5:**
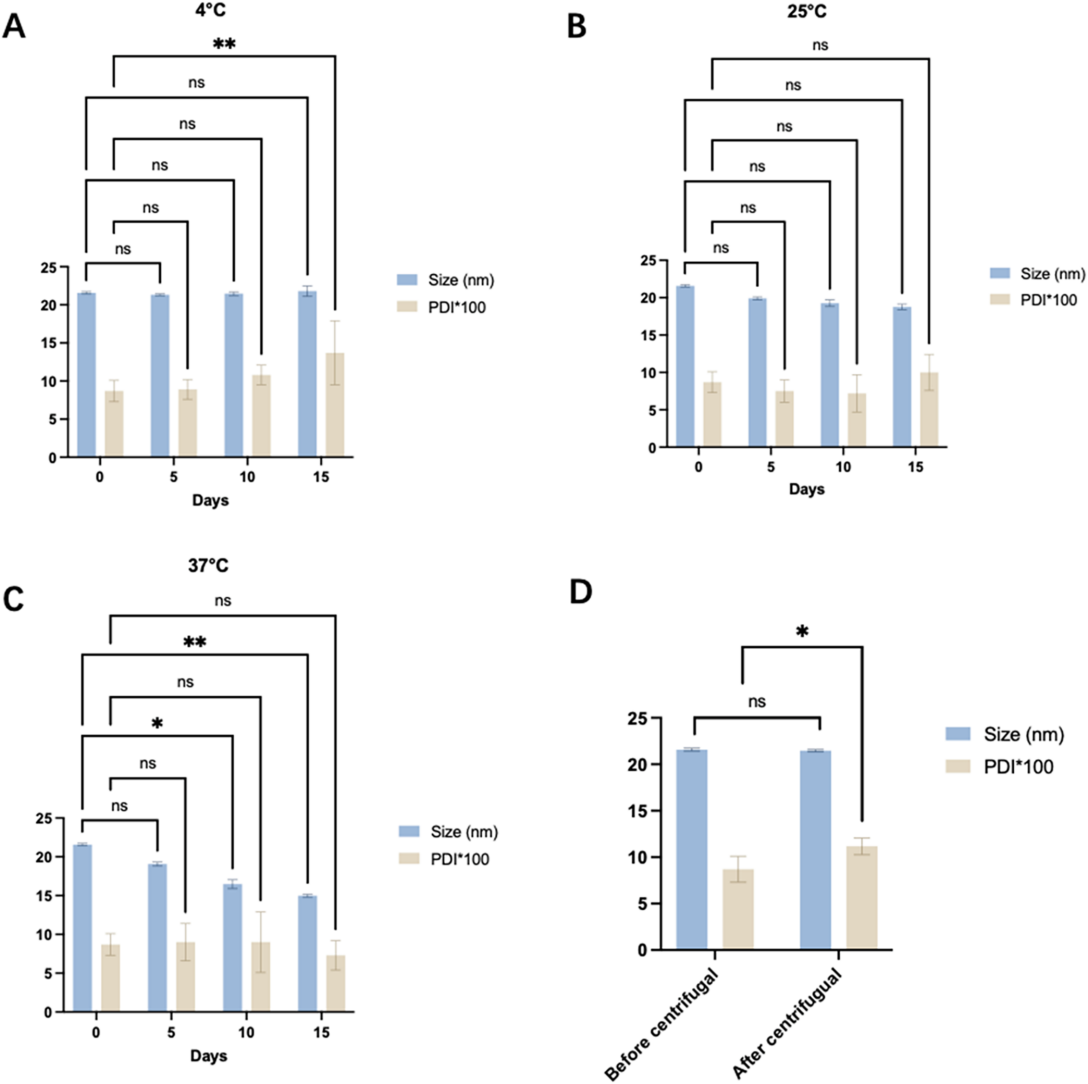
Stability of the FEO-ME. (A) Changes in particle size and PDI of FEO-ME after 15 days of storage at 4 °C. (B) Changes in particle size and PDI of FEO-ME after 15 days of storage at 25 °C. (C) Changes in particle size and PDI of FEO-ME after 15 days of storage at 37 °C. (D) Particle size and PDI of FEO-ME droplets after centrifugation.

#### 3.4.2 Centrifugation Stability

The effects of rotational speed on the mean particle size and PDI of FEO-ME are shown in Figure 5D. No separation or significant differences in particle size or PDI were observed after centrifugation. The results shown in Table 4 indicate a minimal change in the absorbance of FEO-ME before and after centrifugation, with centrifugal stability constants all remaining below 10%. These results indicate that the prepared microemulsions exhibit relatively favorable centrifugal stability.

**Table 4:** Centrifugal Stability Coefficients of Different FEO-ME Batches

batch	*K*_e_
1	1.16%
2	5.88%
3	3.51%

### 3.5 Anti-*E. coli* Activity

#### 3.5.1 Agar Diffusion Test

Anti-*E. coli* effects of FEO-ME were assessed using the agar disc diffusion test. The results demonstrated significant inhibition of *E. coli* growth by both FEO-ME and FEO. The inhibition zone had diameters of 15.3 ± 0.58 mm (Figure 6A-1) for FEO-ME and 15.7 ± 1.53 mm (Figure 6A-2) for FEO, respectively. In contrast, no significant inhibition of *E. coli* growth was observed with the control treatments: LB (Figure 6A-3) and emulsion without FEO (Figure 6A-4). Figure 6A-5 shows the inhibition zone diameter of levofloxacin, the positive control, which was 35.3 ± 1.54 mm.

**Figure 6:**
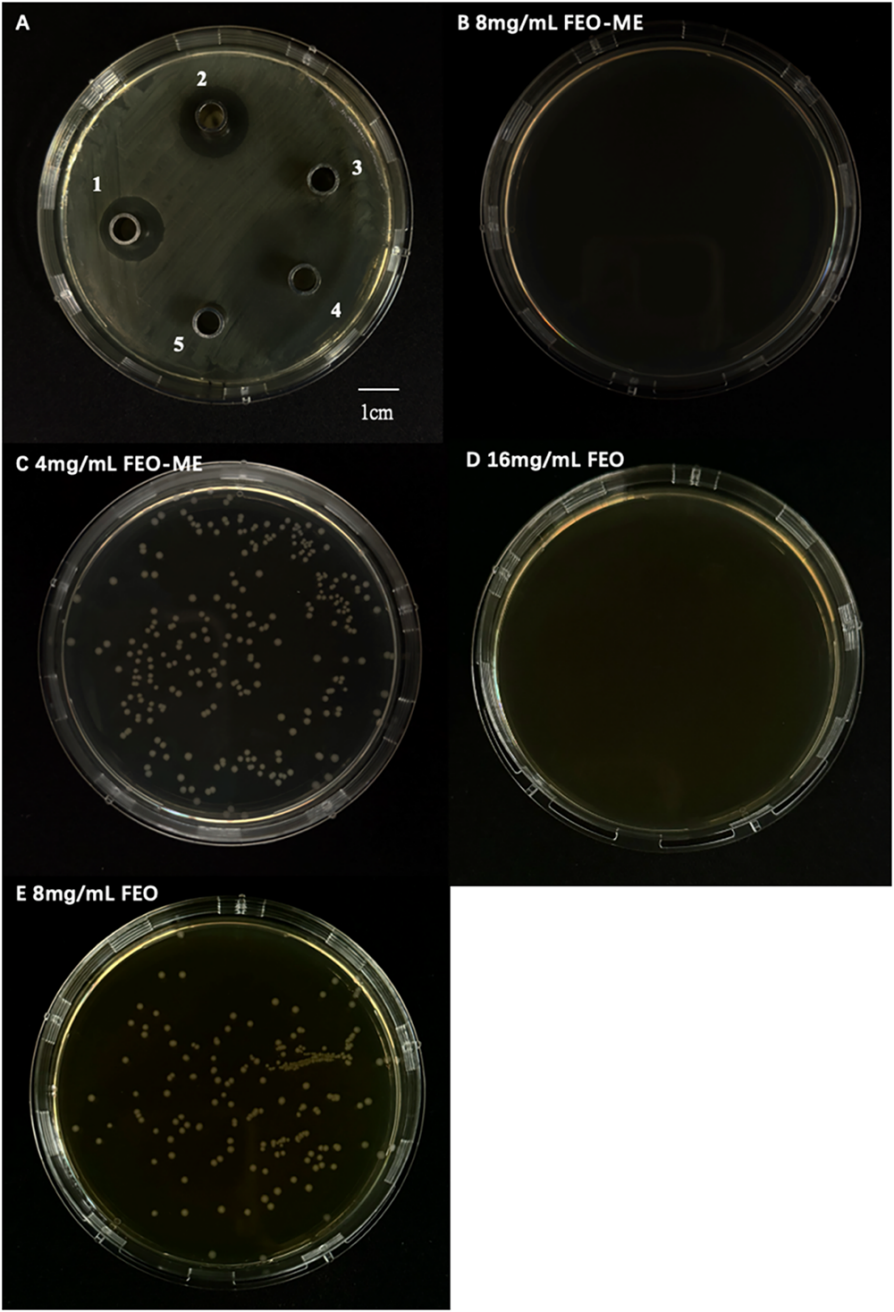
MIC and MBC values in anti-*E. coli*. activity of FEO-ME. (A): Inhibition of *E. coli*. by FEO-ME: A1: FEO-ME; A2: FEO; A3: Blank control (C), A4: Blank microemulsion (C-ME), A5: Positive control (P). Determination of MIC and MBC values of FEO-ME (B, C) and FEO (D, E).

#### 3.5.2 MIC and MBC Values

The MIC and MBC values were determined as shown in Figure 6B–E. At concentrations of 4 mg/mL for FEO-ME and 8 mg/mL for FEO, no visible microbial precipitation was observed in the 96-well plate. However, bacterial growth was detected upon inoculation on agar plates, indicating a minimum inhibitory concentration (MIC). When the concentrations were increased to 8 mg/mL for FEO-ME and 16 mg/mL for FEO, no bacterial growth was observed on the agar plates, suggesting the minimum bactericidal concentration (MBC). This study suggests that microemulsion-encapsulated Forsythia essential oil (FEO-ME) may enhance antimicrobial activity, demonstrating the potential for broad application prospects. In pharmaceuticals, it could serve as a synergistic carrier targeting drug-resistant bacteria when combined with antibiotics. For personal care products, the self-stabilizing nature of microemulsions might improve the transdermal delivery and shelf life of essential oil-based formulations. In food preservation, integrating FEO-ME with biodegradable materials may offer novel approaches to natural antimicrobial packaging. Agriculturally, this technology could support the development of ecofriendly plant protection agents by enhancing environmental resilience of essential oils. These findings highlight the explorative potential of microemulsion systems in functional applications of natural essential oil.

#### 3.5.3 Scanning Electron Microscopy (SEM) Test

As shown in Figure 7, a significant difference in the bacterial morphology of *E. coli* was observed after treatment with FEO-ME or FEO at the MBC concentration for 4 h, compared to untreated bacteria. Specifically, the cell membrane surface of *E. coli* treated with FEO-ME exhibited noticeable damage, while the control group of *E. coli* displayed a smooth surface and retained its characteristic rod-shaped structure. This suggests that FEO-ME exerts its antibacterial effect by damaging the cell wall. These observations further support the potent antibacterial activity of FEO-ME against *E. coli*.

**Figure 7:**
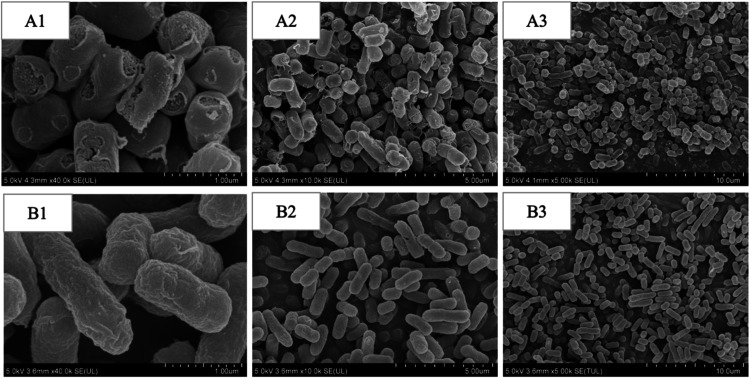
SEM images of *E. coli* treated with FEO-ME and FEO under different magnifications. A1­(1 μm), A2­(5 μm), and A3­(10 μm) were treated with FEO-ME. B1­(1 μm), B2­(5 μm), and B3­(10 μm) were LB control groups without FEO-ME treatment.

## 4 Conclusions

In this study, a stable oil-in-water (O/W) microemulsion of Forsythia essential oil (FEO) with a small droplet size was successfully prepared by using self-microemulsifying systems. Pseudoternary phase diagrams were used to identify distinct formulation regions and determine the optimal and minimal concentrations of the components. Among 12 food-grade surfactant combinations tested, a formulation with a high oil content (10%) and excellent stability was selected. The prepared FEO-ME demonstrated enhanced antibacterial activity against *E. coli* compared to raw essential oil, highlighting the potential of microemulsions to improve the bioactivity of essential oil. These findings provide an effective and novel approach for developing stable, high-oil-content FEO-ME, which could serve as a natural and safe antimicrobial agent.

Further research is needed to explore the broader applications of FEO-ME. Future studies could investigate their antibacterial activity against a wider range of pathogens, including *Staphylococcus aureus* (*S. aureus*) and other foodborne bacteria, to assess their potential in food preservation. Additionally, examining the insecticidal properties of FEO-ME could lead to their use as natural pesticides in agriculture.^45,46^ Their potential for topical applications, such as transdermal absorption and anti-inflammatory effects, also warrants investigation, particularly for pharmaceutical^47^ and cosmetic uses.^48,49^

However, the use of multiple excipients in microemulsion preparation may raise concerns regarding safety and cost. To address these challenges, future research could focus on developing emulsions with smaller droplet sizes that require fewer excipients, enhancing both safety and cost-effectiveness without compromising performance.

In summary, FEO-ME holds great potential in various fields, including food preservation, agriculture, pharmaceuticals, and cosmetics. With further optimization, these microemulsions could serve as powerful tools for enhancing the efficacy and safety of essential oils in diverse applications.
